# A Case of Suspected Cocaine Adulterant-Associated Methemoglobinemia in the United States

**DOI:** 10.7759/cureus.27362

**Published:** 2022-07-27

**Authors:** Nicholas S Dulin, Alexis L Cates, Julia M Cullen

**Affiliations:** 1 Emergency Medicine, Einstein Medical Center Philadelphia, Philadelphia, USA; 2 Emergency Medicine, Bayhealth Hospital, Dover, USA

**Keywords:** lidocaine reaction, local anesthetic, cocaine adulterant, toxicology, methemoglobinemia

## Abstract

A 30-year-old male with a past history of polysubstance use presents to a drug rehabilitation facility after cocaine and heroin use just prior to arrival. While drinking at a water fountain at the facility, he became unresponsive. He was discovered to have an oxygen saturation of 50% on room air with an improvement to 87% on non-rebreather masks. Arterial blood gas revealed a methemoglobin level of 45.8%. The patient was given methylene blue with a repeat methemoglobin level of 0.00% within six hours. We attribute this presentation to local anesthetic-adulterated cocaine, a well-documented cause of methemoglobinemia in the United Kingdom rarely described in the United States.

## Introduction

Methemoglobinemia is defined as a methemoglobin level of >1%, which may result in a range of presentations from asymptomatic to respiratory distress and death. Methemoglobin is produced when the iron atom of a hemoglobin (Hb) molecule loses an electron from its ferrous (Fe2+) state and becomes oxidized to its ferric (Fe3+) state. The remaining binding sites on a methemoglobin molecule will have a greater affinity for oxygen and inhibit oxygen atoms from diffusing into peripheral tissue, secondary to a leftward shift in the oxyhemoglobin dissociation curve. Accumulation of Hb (Fe3+) or methemoglobin is the underlying problem in symptomatic methemoglobinemia, and the cornerstone of management is the removal of oxidant stress (if applicable) and reduction back to hemoglobin using methylene blue. Methemoglobin is also cleared endogenously by the reducing enzyme nicotinamide adenine dinucleotide hemoglobin reductase. However, this enzyme could be overwhelmed by oxidant stressors, in particular local anesthetics, which have been associated with illicit drug use.

Local anesthetics such as benzocaine have been associated with methemoglobinemia [1], with multiple case reports in the literature related to benzocaine-adulterated cocaine. Benzocaine and other local anesthetics are frequently used as an adulterant in cocaine as they are relatively inexpensive and can be used to dilute cocaine and increase profit margins. Local anesthetics will numb mucous membranes and are able to pass rudimentary drug purity tests. Cocaine users may apply cocaine to their oral mucosa with the assumption that less adulterated cocaine will numb the contacted area and indicate higher purity of cocaine. Benzocaine mimics cocaine as they are both ester local anesthetics, inhibit neuron sodium channels from preventing signal transduction, and clinically induce local anesthesia. Benzocaine is much less expensive and more readily available than cocaine which makes benzocaine an ideal cocaine cutting agent. Unfortunately, benzocaine is also a powerful oxidative agent. Benzocaine induces increased oxidation and may lead to the production of methemoglobin 100 to 1000 times the normal rate of metabolism [2]. Methemoglobinemia induced by benzocaine-adulterated cocaine has been described extensively in the literature, particularly in the United Kingdom, for decades. This condition, however, has been described much less frequently in the United States [3-6].

## Case presentation

A 30-year-old male with a past history of polysubstance use presents to a drug rehabilitation facility. He reported prior cocaine use via the intramuscular and gluteal region route, as well as heroin via the intravenous route. The exact time of the patient's last cocaine use prior to presenting at the rehabilitation facility was unknown but reported to be the same day. The patient was asked to provide a urine sample at a rehabilitation facility, drank eight cups of water from a nearby water fountain, and subsequently was found to be unresponsive. He was found by emergency medical servicescyanotic with oxygen saturation (SpO_2_) of 50% on room air, tachypneic, and obtunded. Non-rebreather oxygen was administered, resulting in a SpO_2_ of 87% during transport to the emergency department. The patient remained cyanotic and dusky upon arrival to the ED, still confused but with improving mental status. Additional history obtained from the patient at that time revealed daily alcohol use (approx 1 L vodka/day), daily heroin and cocaine use, as well as benzodiazepine use.

Vital signs revealed a blood pressure of 103/72 mmHg, respiratory rate of 22 per minute, pulse oximetry of 87% on the non-rebreather mask, and a heart rate of 116 beats per minute. Co-oximetry revealed a methemoglobin level of 45.8%. Arterial blood gas revealed a pH of 7.45, partial pressure of carbon dioxide (pCO_2_) of 45.1 mm Hg, a bicarbonate level of 28.9 mmol/L, partial pressure of oxygen (pO_2_) of 301.1 mm Hg with a base excess of 3.7mEq/L. The urine drug screen was positive for cocaine, opiates, and fentanyl. The toxicology screen was significant for undetectable acetaminophen level, undetectable ethanol level, and a subtherapeutic salicylate level of 1.8 mg/dL. Complete blood count revealed a white blood cell count of 10.1x10^9^/L, hemoglobin of 15.0 g/dL, hematocrit of 41.1%, and platelets of 322x10^9^/L. Complete metabolic panel revealed sodium of 133 mEq/L, potassium of 3.1 mEq/L, chloride of 102 mEq/L, bicarbonate of 28 mEq/L, anion gap of 3 mEq/L, blood urea nitrogen of 11 mg/dL, creatinine of 1.1 mg/dL, random glucose of 189 mg/dL, calcium of 9.0 mg/dL, aspartate aminotransferase 14 units/L, alanine aminotransferase 14 units/L, total bilirubin 0.7 mg/dL (Table 1). Coagulation studies revealed a prothrombin time (PT) of 14.1 seconds, activated partial thromboplastin time (aPTT) of 40 seconds, and an international normalized ratio (INR) of 1.21. Additional laboratory studies revealed a troponin T of <0.02 ng/mL, creatine kinase (CK) of 84 U/L, and D-dimer of 1308 µg/mL. Electrocardiogram revealed sinus tachycardia, and a chest X-ray revealed no acute cardiopulmonary abnormalities.

**Table 1:** Laboratory results

Laboratory test	Recorded value	Normal limits
White blood cell count (WBC)	10.1x10^9^/L	4.5 - 11.0 x 109/liter
Hemoglobin (Hgb)	15.0 g/dL	13.0 - 18.0 g/dL
Hematocrit (Hct)	41.10%	37.0 - 49.0%
Platelets (Plt)	322x10^9^/L	130 - 400 x 109/L
Sodium (Na)	133 mEq/L	133 - 145 mEq/L
Potassium (K)	3.1 mEq/L	3.4 - 5.0 mEq/L
Chloride (Cl)	102 mEq/L	95 - 108 mEq/L
Bicarbonate	28 mEq/L	20 - 32 mEq/L
Anion gap	3 mEq/L	6 - 12 mEq/L
Blood urea nitrogen (BUN)	11 mg/dL	8 - 25 mg/dL
Creatinine (Cr)	1.1 mg/dL	0.74 - 1.35 mg/dL
Random glucose	189 mg/dL	70 - 110 mg/dL
Calcium	9.0 mg/dL	8.5 - 10.5 mg/dL
Aspartate aminotransferase (AST)	14 units/L	10 - 40 units/L
Alanine aminotransferase (ALT)	14 units/L	10 - 55 units/L
Total bilirubin	0.7 mg/dL	0.0 - 1.0 mg/dL
Prothrombin time (PT)	14.1 s	11..0 - 13.5 s
Activated partial thromboplastin time (aPTT)	40.0 s	30 - 40 s
International normalized ratio (INR)	1.21	0.8 - 1.1
Troponin T	<0.02 ng/mL	0.0 - 0.01 ng/mL
Creatine kinase (CK)	84 U/L	55 - 170 U/L
D-dimer	1308 µg/mL	0 - 500 µg/mL
Methemoglobin	45.80%	0 - 2.0%
pH	7.45	7.35 - 7.45
Carbon dioxide (pCO_2_)	45.1 mm Hg	35 - 45 mm Hg
Bicarbonate (HCO3)	28.9 mmol/L	22 - 26 mmol/L
Partial pressure of oxygen (pO_2_)	301.1 mm Hg	75 - 100 mmHg
Base excess (BE)	3.7mEq/L	-4.0 - 2.0 mEq/L
Acetaminophen	undetectable	0 - 20 mcg/mL
Ethanol	undetectable	10 - 30mg/dL
Salicylate	1.8 mg/dL	15 - 30 mg/dL

The patient was diagnosed with methemoglobinemia based on the clinical and laboratory findings. The local poison control center was contacted, and toxicology was consulted. Methylene blue was administered intravenously in a bolus dose of 1 mg/kg over 10 minutes. The patient was admitted to the ICU for supplemental oxygen, frequent monitoring, laboratory tests, and repeat methemoglobinemia levels. The patient's cyanosis and mental status improved greatly, and his methemoglobin level six hours after methylene blue administration was undetectable. The patient left against medical advice on hospital day two, and the patient was unable to be reached after multiple attempts to contact him after departure from the hospital.

## Discussion

We hypothesize that this patient's methemoglobinemia was most likely induced by topical anesthetic adulterated cocaine. This suspected case seems to be reported rarely in the United States. Though a polysubstance user, this patient admitted to cocaine use just prior to presenting to rehabilitation with no known novel medication or illicit drug exposures other than heroin and benzodiazepines, which are not associated with methemoglobinemia. Other oxidizing agents commonly implicated in methemoglobinemia include nitrates, nitrites, sulfonamides, phenazopyridine, and dapsone. None of these agents have any known utility as cocaine additives and are therefore very unlikely to account for this patient's methemoglobinemia.

We performed a search of the National Library of Medicine's PubMed with the search terms: "cocaine" and "methemoglobinemia", which yielded 18 results. Only one of these results described a case of methemoglobinemia induced by benzocaine-adulterated cocaine in the United States. This lone case report was described several decades ago, in 1992 [7]. We propose the most likely etiology for this patient's methemoglobinemia is topical anesthetic adulterated cocaine given the timing of onset hours after last cocaine usage, lack of reported additional oxidant stressors with no plausible alternative explanation for co-oximetry confirmed methemoglobinemia. One previous case report utilized urine mass spectrometry to confirm the presence of benzocaine or lidocaine. Unfortunately, this information was unobtainable after the patient left against medical advice, a limitation to our conclusion [4]. A case report from Canada in 2020 of a 49-year-old male who presented with co-oximetry confirmed methemoglobinemia four hours after cocaine use noted the possibility of cocaine adulterants. However, they were also unable to conclude definitively via source cocaine analysis or urine spectrophotometry [8].

## Conclusions

We suspect many cases of adulterant-induced methemoglobinemia may go undiagnosed in the United States due to limitations regarding confirmatory testing, whether from the source of cocaine or from the patient analysis. Further investigation into the true prevalence of this disease is needed. Clinicians should be aware of the possibility of methemoglobinemia in patients who use cocaine and present with cyanosis and hypoxia despite oxygen supplementation, respiratory distress, altered mental status, and other significant clinical features.
